# Does gadoxetate disodium affect MRE measurements in the delayed hepatobiliary phase?

**DOI:** 10.1007/s00330-018-5616-7

**Published:** 2018-07-19

**Authors:** M. Plaikner, C. Kremser, H. Zoller, M. Steurer, B. Glodny, W. Jaschke, B. Henninger

**Affiliations:** 1 Department of Radiology, Medical University of Innsbruck, Anichstraße 35, Innsbruck, Austria; 2 Department of Internal Medicine, Medical University of Innsbruck, Anichstraße 35, Innsbruck, Austria

**Keywords:** Magnetic resonance imaging, Elastography, Liver, Contrast media, Fibrosis

## Abstract

**Objectives:**

To assess if the administration of gadoxetate disodium (Gd-EOB-DTPA) significantly affects hepatic magnetic resonance elastography (MRE) measurements in the delayed hepatobiliary phase (DHBP).

**Methods:**

A total of 47 patients (15 females, 32 males; age range 23–78 years, mean 54.28 years) were assigned to standard hepatic magnetic resonance imaging (MRI) with application of Gd-EOB-DTPA and hepatic MRE. MRE was performed before injection of Gd-EOB-DTPA and after 40–50 min in the DHBP. Liver stiffness values were obtained before and after contrast media application and differences between pre- and post-Gd-EOB-DTPA values were evaluated using a Bland-Altman plot and the Mann-Whitney-Wilcoxon test. In addition, the data were compared with regard to the resulting fibrosis classification.

**Results:**

Mean hepatic stiffness for pre-Gd-EOB-DTPA measurements was 4.01 kPa and post-Gd-EOB-DTPA measurements yielded 3.95 kPa. We found a highly significant individual correlation between pre- and post-Gd-EOB-DTPA stiffness values (Pearson correlation coefficient of r = 0.95 (*p* < 0.001) with no significant difference between the two measurements (*p* =0.49)). Bland-Altman plot did not show a systematic effect for the difference between pre- and post-stiffness measurements (mean difference: 0.06 kPa, SD 0.81). Regarding the classification of fibrosis stages, the overall agreement was 87.23% and the intraclass correlation coefficient was 96.4%, indicating excellent agreement.

**Conclusions:**

Administration of Gd-EOB-DTPA does not significantly influence MRE stiffness measurements of the liver in the DHBP. Therefore, MRE can be performed in the DHBP.

**Key Points:**

*• MRE of the liver can reliably be performed in the delayed hepatobiliary phase.*

*• Gd-EOB-DTPA does not significantly influence MRE stiffness measurements of the liver.*

*• MRE performed in the delayed hepatobiliary-phase is reasonable in patients with reduced liver function.*

## Introduction

Magnetic resonance elastography (MRE) is increasingly used to obtain information regarding tissue stiffness, particularly of the liver, and is comparable to the performance of ‘virtual palpation’ [1]. MRE has been described as an accurate tool for use in hepatic magnetic resonance imaging (MRI) to detect and classify fibrosis in the early stages before morphological changes have occurred [2, 3]. In daily clinical routine, MRE can be performed complementary to diagnostic hepatic MRI. Due to its accurate and non-invasive characteristics for analysing the hepatic parenchyma and lesions, gastroenterologists increasingly request hepatic MRE in addition to standard, contrast-enhanced liver imaging. At our department, MRE has already been integrated into the routine hepatic MRI protocol, depending on the assigned problem.

The liver-specific contrast medium gadoxetate disodium (Gd-EOB-DTPA), also known as Primovist in Europe or Eovist in North America (Bayer Schering Pharma AG), is widely used and has outstanding benefits for liver evaluation [4]. It allows standard dynamic and additional delayed hepatobiliary phase (DHBP) imaging. Gd-EOB-DTPA is taken up by normal hepatocytes, selectively increasing T1 relaxation of normal hepatic tissue in the DHBP and therefore enabling the detection of small tumorous lesions. Thus, liver-specific contrast media have a central role in hepatic MRI with wide acceptance of the prolonged examination time [5]. As Gd-EOB-DTP uptake may be delayed due to a reduced liver function [6], at our department DHBP imaging in patients with clinically suspected reduction in liver function is always performed 40–50 min after the intravenous injection of Gd-EOB-DTPA, as a second, separate examination. During the waiting interval, a short examination with another patient is carried out (e.g. MRI of the knee or the lumbar spine). Many other institutions perform the DHBP 20 min after contrast administration and use the waiting time to perform other sequences that are not significantly influenced by contrast administration, e.g. heavily T2-weighted turbo spin-echo sequences or diffusion-weighted imaging (DWI) [7]. During this waiting time, MRE can also be performed.

The purpose of our study was to assess if Gd-EOB-DTPA administration affects hepatic MRE measurements in DHBP. Therefore, we compared hepatic MRE results for each patient before and after Gd-EOB-DTPA administration.

## Material and methods

### Patients

We retrospectively enrolled 47 patients referred to our department (Department of Radiology, Medical University of Innsbruck) between August 2016 and January 2017 from the gastroenterological department to perform a standardised hepatic MRI, including Gd-EOB-DTPA administration with DHBP imaging and hepatic MRE. Inclusion criteria were a minimum age of 18 years as well as an entirely performed hepatic MRI protocol (Table 1), especially including hepatic MRE performed both before contrast media injection and after 40–50 min in DHBP. Because of the retrospective nature of this study, institutional review board approval was granted by means of a general waiver (Local Research Ethics Committee, Medical University of Innsbruck; 20 February 2009).

**Table 1:** Succession of sequences used for the hepatic MRI protocol

Temporal numbering	Designation of the sequence
1	Transverse T2w Half-Fourier-Acquired Single-shot Turbo Spin-Echo (HASTE)
2	Transverse spin-echo-based diffusion-weighted echo planar imaging (SE-DWI)
3	Transverse T1 3D Multiecho Dixon VIBE (T1 VIBE q-dixon)
4	MRE (3 slices)
5	Transverse volume-interpolated breath-hold examination (VIBE)-DIXON (native)
6	Gd-EOB-DTPA injection + Dynamic VIBE Controlled Aliasing In Parallel Imaging Results In Higher Acceleration Factor (CAIPIRINHA)-DIXON-TWIST (late arterial) with 3 datasets
7	Transverse VIBE-DIXON (venous, ~45 s after injection)
8	Transverse VIBE-DIXON (delayed, ~2 min after injection)
9	Transverse fat saturated T2w TSE
10	Transverse VIBE-DIXON (late, ~7 min after injection)
11	40- to 50-min waiting period (from the injection) outside the scanner, patient is repositioned after this period
12	Transverse VIBE-DIXON (in the DHBP)
13	Coronal VIBE-DIXON (in the DHBP)
14	MRE (3 slices)

### MRI

MRI was performed in the supine position using a 1.5T MRI scanner (Magnetom AvantoFit, Siemens Healthineers) with an 18-channel body phased-array surface coil. The succession of sequences used for our hepatic MRI protocol is shown in Tables 1 and 2, along with the respective imaging parameters.

**Table 2:** Imaging parameters of different MRI sequences

	T2w HASTE	SE-DWI	T1 VIBE q-DIXON	VIBE-DIXON	MRE sequence	Dynamic VIBE CAIPIRINHA-DIXON-TWIST	T2w TSE
Type	2D	2D	3D	3D	2D	3D	2D
Orientation	Transverse	Transverse	Transverse	Transverse / coronal	Transverse	Transverse	Transverse
FOV (mm)	380	380	360	380	420	380	380
Slice thickness (mm)	6	6	3.5	3	5	3	5
Slice gap (mm)	1	1.2	0	0	-	0	1
Slices	35	31	64	72	1	72	35
Acquisition matrix	320 x 256	134 x 120	160 x 111	320 x 195	128 x 103	288 x 174	384 x 216
Parallel imaging factor	2	2	3	4	2	4	2
TR (ms)	1200	3500	15.6	6.68	50	6.32	7725
TE (ms)	93	59	2.38/4.76/7.14/9.52/11.9/14.28	2.39 / 4.77	23.75	2.39 / 4.77	96
Flip angle (°)	90	90	4	10	25	10	90
Echo train length	102	120	6	2	1	2	18
Fat saturation	no	Frequency selective	DIXON	DIXON	no	DIXON	Frequency selective
b-values	-	50 / 400 / 1000	-	-	-	-	-
Averages	1	1 / 2 / 2	1	1	1	1	2
Acquisition time (min)	1:45	3:32	0:19	0:14	0:05	0:07	3:57

Patients received 10 ml of Gd-EOB-DTPA via manual injection through a peripheral intravenous line inserted at the cubital fossa or forearm, with an approximate injection rate of 1.5–2.0 ml/s, followed by a 20-ml saline flush. Hepatic MRE was performed before and 40–50 min after Gd-EOB-DTPA administration (i.e. in DHBP). Mechanical 60-Hz vibrations were applied using a circular passive acoustic driver (Resoundant, Inc.) placed against the right abdominal wall and fixed using an elastic band. A two-dimensional commercial phase-contrast gradient-echo (GRE) sequence as provided by the manufacturer was applied, which performs inline calculation of wave and stiffness images together with a 95% confidence map showing areas with less reliable stiffness crossed out [8]. For every patient, three transverse slices through the liver were acquired, with one slice located in the cranial, one in the middle (portal vein at the level of the hilus) and one in the caudal hepatic section.

To assess the hepatic iron state a commercially available 3D multi-echo gradient (GRE) sequence (t1 vibe q-dixon) was used [9].

### Data analysis

For each patient, all three transverse hepatic MRE slices before and after Gd-EOB-DTPA administration were evaluated. Stiffness values were reported in kPa units. An as large as possible polygonal region of interest (ROI) was selected in the liver parenchyma of every transverse section within the 95% confidence region of the acquired stiffness maps. Manual ROI placement was carefully performed by one radiologist with over 6 years’ experience in liver imaging (PM) examining wave images for proper wave propagation and using the magnitude image to avoid major vessels, liver surface and artefacts (especially movement). For further analysis, the mean values of the three polygonal ROIs were used. Patients were classified by the authors into four fibrosis stages according to their mean stiffness value, based on values from the actual literature [10, 11]: F0 = 0–2.5 kPa, F1 = 2.5–3 kPa, F2 = 3–4.4 kPa, F3 = 4.4–7 kPa, and F4 > 7 kPa. Hepatic R2* values were obtained from R2* maps provided by the t1 vibe q-dixon sequence at identical slice positions and checked for iron overload [12] based on the classification recommended by the EASL International Consensus Conference on Haemochromatosis [13].

### Statistical analysis

Statistical calculations were performed using the R Project for Statistical Computing [14]. The Shapiro–Wilk normality test was used to assess the normal distribution of the given population. Because data were not normally distributed, the Mann–Whitney–Wilcoxon test was performed to test for a significant difference between pre- and post-Gd-EOB-DTPA MRE data. Results were considered significant at *p*-values of <0.05. To determine the correlation between pre- and post-Gd-EOB-DTPA MRE data, Pearson’s correlation coefficient was calculated, and the data were compared using a Bland–Altman plot. To determine the agreement between fibrosis staging data before and after Gd-EOB-DTPA administration, contingency tables were created; Pearson’s chi-squared test was performed, and overall agreement, as well as the Cohen’s Kappa coefficient with equal weights and the two-way intraclass correlation coefficient was calculated using the irr-package for R [15].

## Results

A total of 47 patients (15 females and 32 males; age range, 23–78 years; mean age, 54.28 years) were enrolled in this study. All patients had the clinical suspicion of impaired liver function: 14/47 patients were transferred post-liver transplantation, 14/47 had unclear, suspicious liver lesions and 19/47 patients suffered from diffuse liver injury including cirrhosis, fibrosis, iron overload or primary sclerosing cholangitis. Of all patients only one was found to have minimal iron overload (R2* of 102 1/s).

**Fig. 1:**
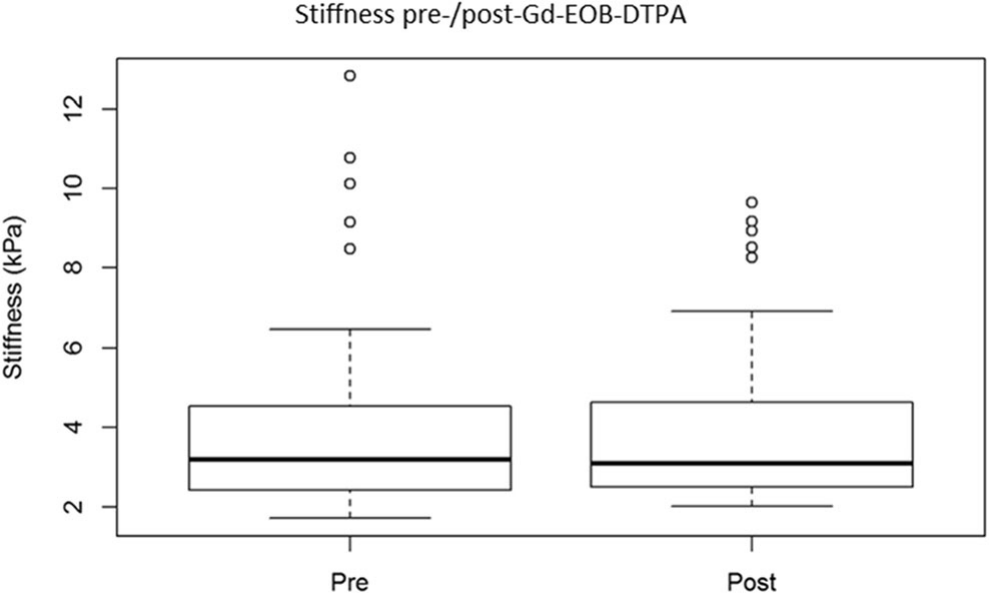
Hepatic MRE mean stiffness values for all pre- (**left**) and post- (**right**) Gd-EOB-DTPA ROI measurements in kPa

The mean area of all polygonal ROIs was 48.18 cm^2^ (range, 12.05–155.23 cm^2^). The mean hepatic stiffness values of all pre- and post-Gd-EOB-DTPA ROI measurements were 4.01 kPa (1.73–12.81, median 3.20) and 3.95 kPa (2.03–9.65, median 3.10), respectively (Fig. 1).

**Fig. 2:**
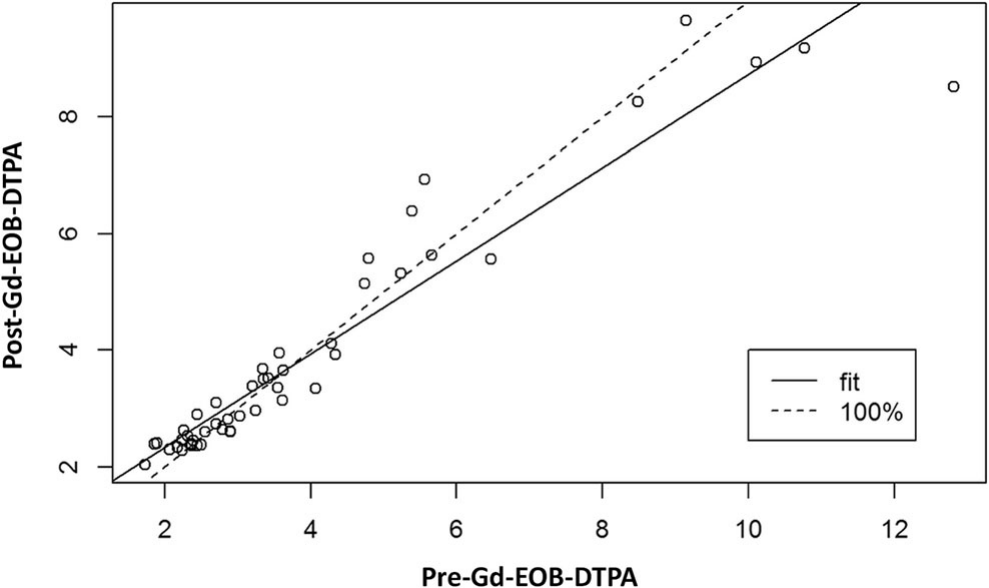
Correlation between pre- and post-Gd-EOB-DTPA hepatic MRE measurements (kPa). The continuous line represents the best linear fit, and the dotted line represents 100% agreement. The Pearson correlation coefficient was 0.95 (*p* < 0.001)

**Fig. 3:**
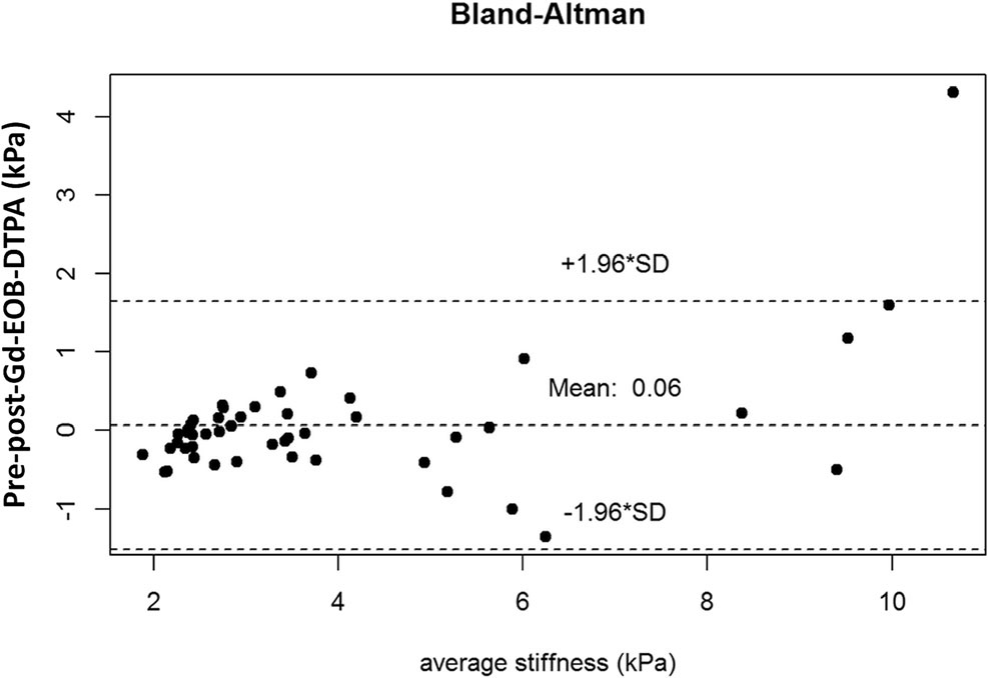
Bland–Altman plot for all mean hepatic MRE stiffness measurements (kPa). No systematic effect was observed in the difference between pre- and post-values (mean difference, 0.06 kPa; standard deviation, 0.81)

The individual correlation between pre- and post-Gd-EOB-DTPA stiffness values is shown in Fig. 2 (Pearson correlation coefficient of r = 0.95; *p* < 0.001). The Bland–Altman plot (Fig. 3) did not show a systematic effect in the difference between pre- and post-stiffness measurements (mean difference, 0.06 kPa; standard deviation [SD], 0.81; range, −1.36 to 4.30). The Mann–Whitney–Wilcoxon test showed no significant differences between pre- and post-Gd-EOB-DTPA hepatic stiffness values (*p* = 0.49).

The classification of patients according to their mean stiffness values into different fibrosis stages led to the following results for pre-Gd-EOB-DTPA measurements: 15 patients were classified as stage F0 (normal hepatic tissue); seven as stage F1; 13, stage F2; seven, stage F3; and five, stage F4. Regarding the post-Gd-EOB-DTPA data, 12 patients were classified as stage F0; 11, stage F1; 12, stage F2; seven, stage F3; and five, stage F4. The resulting contingency table is presented as Table 3. The overall agreement was 87.23%; Cohen’s kappa was 0.84. The Pearson chi-squared test was highly significant (*p* < 0.001) and the two-way intraclass correlation coefficient was 0.96 (95% confidence interval, 93.7–98), indicating excellent agreement. Overall, the obtained fibrosis stage deviated between pre- and post-Gd-EOB-DTPA measurements in only six patients. The individual mean stiffness values for these patients are listed in Table 4. In all cases, the mean stiffness value was near the range limit of the respective fibrosis stage, and the mean difference between pre- and post-stiffness values was −0.16 kPa (range: -0.44 – 0.29).

**Table 3:** Contingency table for the agreement between obtained classifications into fibrosis groups (F0–F4) based on pre- and post-Gd-EOB-DTPA stiffness measurements

	Post-Gd-EOB-DTPA					
	Fibrosis stage (F)	0	1	2	3	4
Pre-Gd-EOB-DTPA	0	12	3	0	0	0
	1	0	6	1	0	0
	2	0	2	11	0	0
	3	0	0	0	7	0
	4	0	0	0	0	5

Rows represent the classification based on pre-Gd-EOB-DTPA stiffness measurements, and columns indicate the classification based on post-Gd-EOB-DTPA data

**Table 4:** Individual mean stiffness values for patients with different resulting fibrosis classifications obtained based on pre- and post-Gd-EOB-DTPA stiffness measurements (kPa)

Patient (no.)	Fibrosis stage pre-Gd-EOB-DTPA	Fibrosis stage post-Gd-EOB-DTPA	Mean stiffness pre-Gd-EOB-DTPA	Mean stiffness post-Gd-EOB-DTPA	Stiffness difference
1	2	1	3.025	2.861	0.164
2	1	2	2.703	3.099	−0.396
3	0	1	2.441	2.884	−0.443
4	2	1	3.249	2.957	0.292
5	0	1	2.265	2.616	−0.351
6	0	1	2.318	2.529	−0.210

**Fig. 4:**
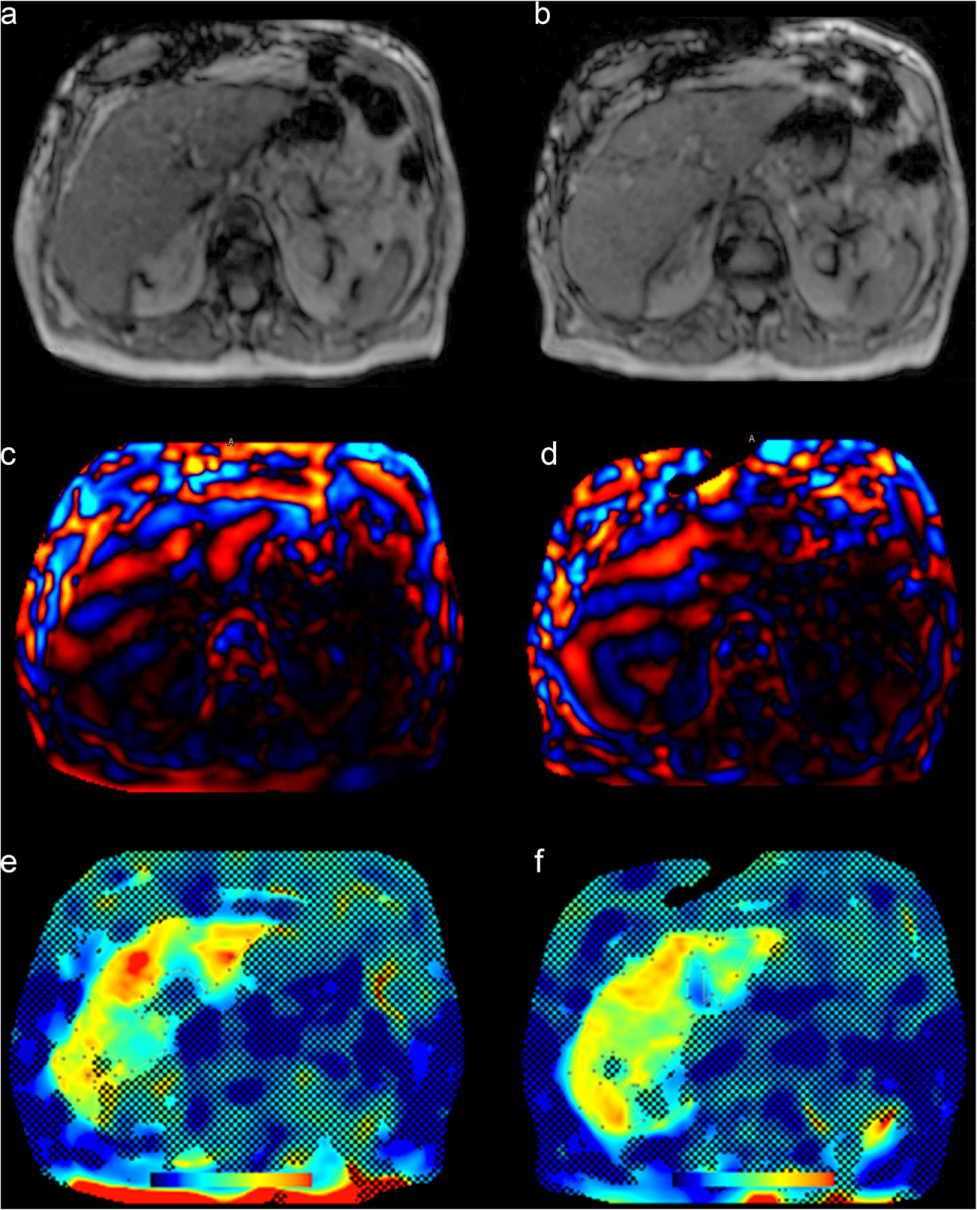
Example of hepatic MRE with stiffness measurements performed before (**e**) and after (**f**) Gd-EOB-DTPA administration together with the respective magnitude (**a, b**) and wave (**c, d**) images. The resulting mean hepatic stiffness value for (**e**) was 4.98 kPa and (**f**) was 4.83 kPa, both indicating fibrosis grade F3. The dotted line represents the measured area of the liver parenchyma within the 95% confidence map

**Fig. 5:**
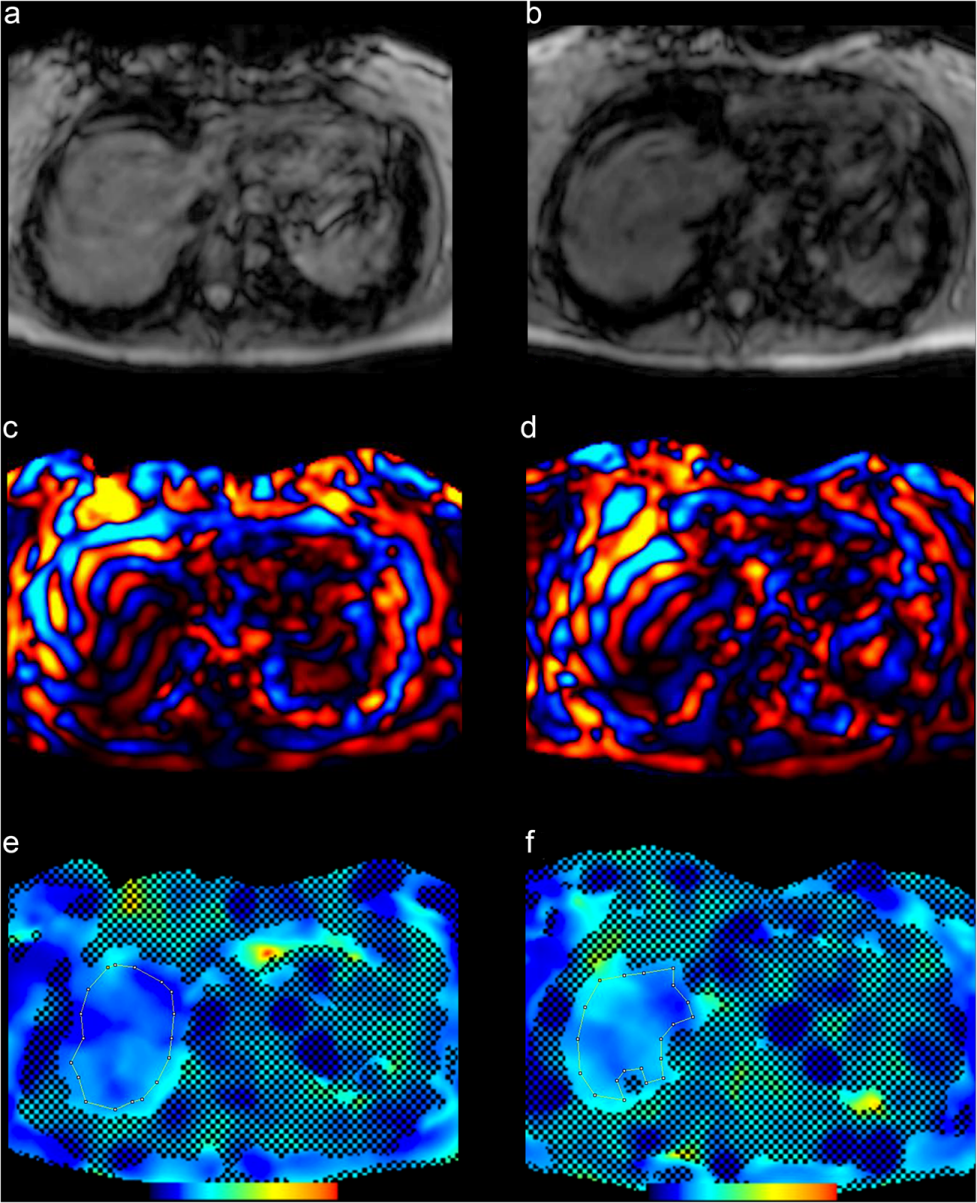
Hepatic MRE performed before (**e**) and after (**f**) Gd-EOB-DTPA administration. Corresponding magnitude (**a, b**) and wave (**c, d**) images are provided. Normal stiffness values were obtained with both measurements (**a**: 1.87 kPa and **b**: 1.60 kPa). The dotted line represents the measured area of the liver parenchyma within the 95% confidence map

Typical exemplary stiffness maps are shown in Figs. 4 and 5 together with corresponding magnitude and wave images.

## Discussion

Hepatic MRI with Gd-EOB-DTPA administration is a standard imaging procedure for the assessment of various hepatic conditions [16]. The additional benefits of hepatic MRE attracts increasing interest, and several centres have already included MRE into the routine hepatic MRI protocol to evaluate diffuse liver disease and characterise focal lesions [17].

Our results indicate a high, significant correlation between pre- and post-Gd-EOB-DTPA stiffness values (Pearson correlation coefficient of r = 0.95; *p* < 0.001), with no significant differences between the two values (*p* = 0.49). Furthermore, no systematic effect was observed for the difference between pre- and post-stiffness measurements (mean difference, 0.06 kPa; SD, 0.81) using the Bland–Altman plot.

In our study, the classification of patients into fibrosis stages indicated an excellent agreement between pre- and post-MRE measurements. Only six of the 47 patients displayed a different assignment on comparing the pre- and post-Gd-EOB-DTPA stiffness measurements. In these cases, the values were very close to the defined cut-off level between two stages, with a maximum absolute difference of stiffness values of only 0.44 kPa. A possible reason for these overall small differences could be slightly different slice locations of the transverse sections through the liver after repositioning the patient for the second examination. In this context a possible influence due to a slightly different driver position could also be discussed [18].

To the best of our knowledge, this is the first study to evaluate the effect of liver-specific contrast medium administration on hepatic MRE stiffness measurements performed before and after (40–50 min) the administration in the DHBP. By this time, Gd-EOB-DTPA has entirely been taken up by the hepatocytes, increasing the T1 relaxation and raising the normal liver signal intensity, which may theoretically influence the stiffness measurements [19]. Halinan et al reported that intravenous gadolinium-diethylenetriamine penta-acetic acid (Gd-DTPA) has no significant influence on liver stiffness measurements using hepatic MRE and does not significantly affect the diagnostic performance of hepatic MRE for fibrosis detection [20]. As opposed to our study, the authors did not use a liver-specific contrast medium and performed hepatic MRE measurements before and 5 min after intravenous contrast medium injection. Motosugi et al hypothesised that the phase shift of the contrast medium does not affect the stiffness measurements obtained using hepatic MRE [21] and demonstrated the lack of influence of Gd-EOB-DTPA on hepatic MRE stiffness measurements in their study. However, their post-contrast medium measurements were performed 20 min after Gd-EOB-DTPA administration, with the patient remaining on the scanning table between the two hepatic MRE acquisitions.

Owing to its structure, Gd-EOB-DTPA is rapidly absorbed at a high dose by hepatocytes, leading to better visualisation of normal hepatic tissue through parenchymal enhancement [22]. Some studies have indicated that without reaching statistical significance, Gd-EOB-DTPA can influence DWI and T2-weighted images by causing higher magnetic susceptibility and T2 shortening [23, 24]. In general, the DHBP with Gd-EOB-DTPA is acquired about 20 min after contrast administration, because at this time normal hepatocytes reach maximum signal intensity [25]. However, the time of contrast uptake may differ between individuals because the hepatic elimination pathway is related to hepatocyte function [26]. Studies indicate that enhancement of liver parenchyma is best at 20 min after administration of Gd-EOB-DTPA followed by a signal intensity plateau for at least 2 h [27, 28]. It is known that the Gd-EOB-DTPA transport correlates with liver function and therefore the peak of Gd-EOB-DTPA accumulation in a damaged liver is delayed [29]. Thus, in compromised liver parenchyma (e.g. fibrotic or cirrhotic) the hepatobiliary phase may be delayed due to the reduced liver function [6, 30, 31]. For this reason, at our centre, whenever liver MRI is performed on patients with clinical suspicion of impaired liver function, a second imaging session with new patient positioning in the scanner is performed 40–50 min after the first examination. The idea behind the present study was to assess if MRE could be performed during this second session. In this case the initial session could be kept relatively short and the MRE driver would not have to be applied during this session, possibly leading to easier breathing and breath-holding for patients. This is relevant to well-known problems of arterial phase imaging with Gd-EOB-DTPA, which has been recently associated with acute transient dyspnoea [32, 33]. The hepatic protocol is an exhausting examination for patients, especially when their general condition is restricted. Therefore, the MRI examination should be performed as pleasantly as possible, with the best possible image quality.

For MRE, motion encoding gradients are used, leading to prolonged echo times and consequently to decreased image quality in case of T2 or T2* reduction, as for example is the case for iron overload [34]. Therefore, especially when based on GRE sequences, increased iron levels are often a limiting factor for MRE [35]. As contrast agents lead to a T2 and T2* reduction, these effects might likewise be a limiting factor for MRE quality. However, in contrast to iron, for Gd-EOB-DTPA the corresponding T1 effect dominates, because T1 and T2 relaxivities are of approximately the same magnitude [36]. Therefore, for our MRE sequence, the T1 effect of the contrast agent compensates any signal decrease due to the simultaneous T2 and T2* shortening, and the image quality with and without contrast agent is comparable or even tends to be superior after contrast administration. In our patient cohort only one patient showed slight iron overload and no notable decrease in image quality could be found. Our objective was to evaluate liver stiffness values before and after contrast administration so that even if iron was present, its effect remained the same in both sessions. When dealing with patients suffering from iron overload, spin echo-based echo planar imaging, fast spin echo methods or T1-based tagged MRI are more reliable methods than GRE sequences as used in our study [37, 38].

Our study has some limitations that must be addressed. First, our study population was rather small compared with those of other studies. Furthermore, some conditions, e.g. inflammation, congestion, portal hypertension and cholestasis, can also cause increased liver stiffness [39]. Kim et al [40] illustrated in their recent paper that liver stiffness measured by MRE increases as cholestasis increases. This effect was not ruled out in our study, so it might be possible that in patients with modest biliary obstruction administration of Gd-EOB-DTPA might cause a transient rise in liver stiffness. Another drawback is that no histological correlation was available; however, the purpose of our study was not to evaluate whether hepatic MRE findings correlated with clinical or histological findings (which have been assessed in several other studies [41, 42]), but to compare the results within individuals’ data. Furthermore, it was not possible to prove that MRI without passive driver administration really improves the quality of the examination because our study did not include MRI examinations without a passive driver. Hence, this statement remains a hypothesis that needs to be tested in another study. We used a two-dimensional phase-contrast GRE sequence to gather three transverse slices through the liver parenchyma and manually positioned an as large as possible polygonal ROI within the 95% confidence region of the acquired stiffness maps. This generally accepted method [43] allows the evaluation of a large portion, but not of the entire organ, because usually the 95% confidence region with reliable stiffness values is smaller than the entire liver [8]. The use of true three-dimensional imaging could be alternatively discussed, even though longer acquisition times might be problematic for breath-holding [18], and these methods are not yet commercially available. Technical details to improve image quality need to be evaluated in future studies. Finally, inter- and intra-observer variability was not evaluated in our study; however, reproducibility and repeatability of MRE measurements for staging of liver fibrosis were already studied in detail by Lee et al [44].

In conclusion, Gd-EOB-DTPA administration does not significantly influence hepatic MRE stiffness measurements in the DHBP, 40–50 min after contrast medium administration. Therefore, for daily clinical routine, hepatic MRE measurements can reliably be performed at any time in the DHBP.

## Abbreviations

DHBP: Delayed hepatobiliary phase
F0-4: Fibrosis stage 0-4
Gd-EOB-DTPA: Gadoxetate disodium
kPa: Kilopascal
min: Minutes
ml/s: Millilitre/second
MRE: Magnetic resonance elastography
MRI: Magnetic resonance imaging
ROI: Region of interest
SD: Standard deviation
T: Tesla
